# Pseudo-Starvation Driven Energy Expenditure Negatively Affects Ovarian Follicle Development

**DOI:** 10.3390/ijms22073557

**Published:** 2021-03-30

**Authors:** Li Meng, Verena Coleman, Yu Zhao, Mario Ost, Anja Voigt, Annelies Bunschoten, Jaap Keijer, Katja Teerds, Susanne Klaus

**Affiliations:** 1 National Engineering Research Center for Breeding Swine Industry, College of Animal Science, South China Agricultural University, Guangzhou 510642, China; limeng@scau.edu.cn; 2 Human and Animal Physiology, Wageningen University, De Elst 1, 6708 WD Wageningen, The Netherlands; shzhaoyu0523@sina.com (Y.Z.); annelies.bunschoten@wur.nl (A.B.); jaap.keijer@wur.nl (J.K.); 3 German Institute of Human Nutrition in Potsdam-Rehbruecke, Arthur-Scheunert-Allee 114-116, 14558 Nuthetal, Germany; verena.coleman@dife.de (V.C.); mario.ost@dife.de (M.O.); anja.voigt@dife.de (A.V.); 4 Institute of Nutritional Science, University of Potsdam, 14469 Potsdam, Germany

**Keywords:** ovarian follicular development, IGF1, energy metabolism, UCP1, IRS1, AKT

## Abstract

In the present investigation, we examined whether a change in whole body energy fluxes could affect ovarian follicular development, employing mice ectopically expressing uncoupling protein 1 in skeletal muscle (UCP1-TG). Female UCP1-TG and wild-type (WT) mice were dissected at the age of 12 weeks. Energy intake and expenditure, activity, body weight and length, and body composition were measured. Plasma insulin, glucose, leptin, plasma fibroblast growth factor 21 (FGF21) and plasma insulin-like growth factor 1 (IGF1) levels were analyzed and ovarian follicle and corpus luteum numbers were counted. IGF1 signaling was analyzed by immunohistochemical staining for the activation of insulin receptor substrate 1/2 (IRS1/2) and AKT. UCP1-TG female mice had increased energy expenditure, reduced body size, maintained adiposity, and decreased IGF1 concentrations compared to their WT littermates, while preantral and antral follicle numbers were reduced by 40% and 60%, respectively. Corpora lutea were absent in 40% of the ovaries of UCP1-TG mice. Phospho-IRS1, phospho-AKT -Ser473 and -Thr308 immunostaining was present in the granulosa cells of antral follicles in WT ovaries, but faint to absent in the antral follicles of UCP1-TG mice. In conclusion, the reduction in circulating IGF1 levels due to the ectopic expression of UCP1 is associated with reduced immunostaining of the IRS1-PI3/AKT pathway, which may negatively affect ovarian follicle development and ovulation.

## 1. Introduction

Female reproduction, including folliculogenesis, ovulation, fertilization, embryo development, parturition, and lactation, is one of the most energy-costly biological processes that a female mammal can undertake [1] and is proposed to be a driving force in evolution [2]. Reproduction can only be successful when sufficient energy resources guarantee the metabolic demands imposed by pregnancy and, especially, lactation [3,4]. Not surprisingly, there exists a close relation between female reproduction and whole-body energy status. Accordingly, studies in rodents have shown that caloric restriction (CR) without malnutrition starting at weaning results in a delayed puberty onset, reduced fertility and smaller litter sizes [5,6]. CR is an intervention that reduces energy intake, resulting in decreased adiposity, increased metabolic rate and decreased availability of oxidizable energy (fuel) [7]. It has been suggested that particularly the reduced fuel availability is responsible for the observed negative effects on fecundity [8]. This agrees with the rapid restoration of fertility upon return to ad libitum feeding [9,10], which occurs before restoration of adiposity [9].

The uncoupling protein 1 transgene (UCP1-TG) mouse specifically overexpresses UCP1 in skeletal muscle. This mouse serves as a model of skeletal muscle (SKM) pseudo-starvation, displaying an increased muscle energy and substrate demand [11,12], leading to a reallocation of fuel availability. UCP1 is located in the inner mitochondrial membrane and is normally expressed in brown adipose tissue, though under specific conditions its expression can be induced in white adipose tissue [13]. UCP1 can be activated by adrenergic stimuli and low ambient temperature, causing the dissipation of the proton gradient across the mitochondrial inner membrane, uncoupling the respiratory chain from ATP synthesis [14]. The ectopic expression of UCP1 in SKM increases energy expenditure and imposes a condition of mild energy insufficiency [11,12,15]. SKM UCP1-TG mice display decreased circulating insulin-like growth factor 1 (IGF1) concentrations, despite having increased growth hormone levels. IGF1, which is primarily synthesized in the liver, circulates tightly bound to IGF1 binding proteins [16]. IGF1 and its binding proteins play an important role in ageing and in the pathogenesis of several common types of cancers. Next to the liver, IGF1 is also produced locally in a number of other tissues including the ovary, where it is involved in growth hormone independent autocrine/paracrine functions. IGF1 primarily acts by binding to and activating the IGF1 receptor 1 (IGF1R) [16]. This receptor is widely distributed and enables IGF1 to coordinate balanced growth among multiple tissues and organs such as the ovary [17,18,19]. In the ovary, IGF1R is specifically expressed in the granulosa cells of healthy growing follicles [20,21,22,23]. Female mice lacking IGF1 are infertile, with follicular development being arrested at the small antral stage [24,25]. In vitro studies have shown that IGF1 can augment the action of the gonadotropic hormone follicle-stimulating hormone (FSH) on granulosa cell proliferation to ensure follicular survival [25,26,27]. IGF1 promotes cell growth and suppresses the apoptosis of granulosa cells by activating the phosphatidylinositol 3-kinase PI3K/AKT pathway [27,28,29]. Indeed, IGF1R inactivation reduces follicle growth, possibly by decreasing the activity of phosphorylated AKT [27]. IGF1 signaling in the ovary was initially thought to be mediated by insulin receptor substrate 2 (IRS2), as the deletion of the *Irs2* gene caused infertility [30]. Recent in vitro studies have shown, however, that the activation of the PI3K/AKT pathway in granulosa cells is a consequence of the convergence of IGF1 and FSH signaling pathways, facilitating the phosphorylation of IRS1 and subsequent activation of PI3K/AKT [31]. Whether under in vivo conditions IGF1 signaling makes use of activation of both IRS1 and IRS2 is at present unresolved.

While in particular male UCP1-TG mice have been investigated extensively metabolically [15,32,33,34,35,36], female UCP1-TG mice also display increased energy expenditure and energy intake [12], making this a suitable model to examine the effects of a peripheral energy drain on ovarian follicular development. We therefore aim to investigate the effects of UCP1 overexpression in SKM, on ovarian follicular reserve and ovulation rate. We especially address the role of IGF1, as plasma concentrations of this fertility-related peptide hormone are strongly reduced in UCP1-TG male mice [37]. We hypothesize that peripheral IGF1 concentrations will be severely reduced in the UCP1-TG female mice, which will lead to reduced follicular development, a process that seems to be associated with decreased activation of the IRS1-PI3K/AKT pathway.

## 2. Results

### 2.1. Morphometric Measurements

**Figure 1:**
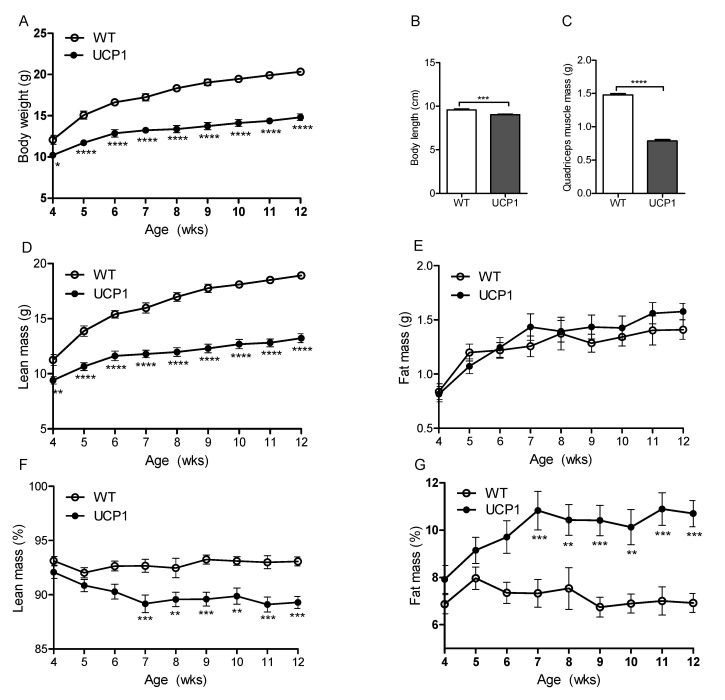
Morphometric data of female mice overexpressing UCP1 in skeletal muscle (UCP1-TG; UCP1) and wild-type (WT) littermate controls, both fed chow diets for 12 weeks. Body weight development (**A**), body length at week 12 of age (**B**), quadriceps weight at the age of 12 weeks (**C**), lean body mass development (**D**), body fat mass development (**E**), lean body mass as percentage of body mass (**F**) body fat mass as percentage of body mass (**G**) were determined. Statistical analyses were carried out based on 8 mice per group, except for body length and quadriceps weight (*n* = 10). Data are expressed as mean +/− SEM; (**A**,**D**,**E**,**F**,**G**): two-way ANOVA; (**B**,**C**): Student’s t-test; * *p* < 0.05, ** *p* < 0.01, *** *p* < 0.001, **** *p* < 0.0001.

The body weight of female UCP1-TG mice was significantly lower than of WT littermates from week 4 onwards up to the age of 12 weeks, when the animals were sacrificed (Figure 1A). Concomitantly, UCP1-TG mice showed a decreased body length (Figure 1B), quadriceps muscle mass (Figure 1C), and lean body mass (Figure 1D), while total body fat mass was not different (Figure 1E) compared to WT littermates. Consequently, lean body mass percentage was decreased (Figure 1F), while the percentage of body fat mass was increased (Figure 1G) in the UCP1-TG mice from week 7 onwards compared to WT littermate controls.

### 2.2. Energy Metabolism

**Figure 2:**
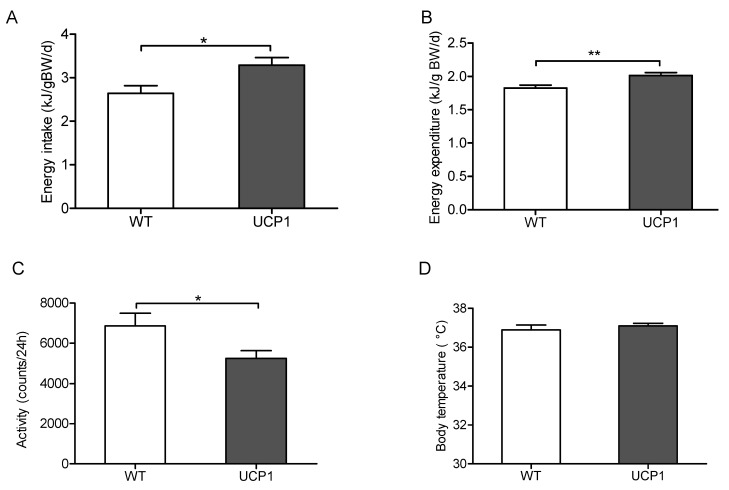
Energy intake (**A**), energy expenditure (**B**), activity counts over 24 h (**C**) and body temperature (**D**) in 12-week-old UCP1-TG (UCP1) and wild-type (WT) littermate controls fed a chow diet. Data are expressed as mean +/− SEM (*n* = 8); (**A**–**D**): Student’s t-test; * *p* < 0.05, ** *p* < 0.01.

Energy intake, energy expenditure and physical activity were measured by indirect calorimetry at the age of 11 weeks. In the UCP1-TG mice, energy intake and energy expenditure were significantly increased, which was accompanied by decreased physical activity when compared to WT mice (Figure 2A–C). These results suggested that the increased energy expenditure was not due to increased physical activity but resulted from UCP1 overexpression in SKM. The combined increase in energy expenditure and energy intake further indicated an increased substrate flux towards SKM, as previously shown in male mice [32,33]. There were no differences in body temperature between UCP1-TG and WT mice (Figure 2D).

### 2.3. Plasma Glucose and Hormone Concentrations

**Figure 3:**
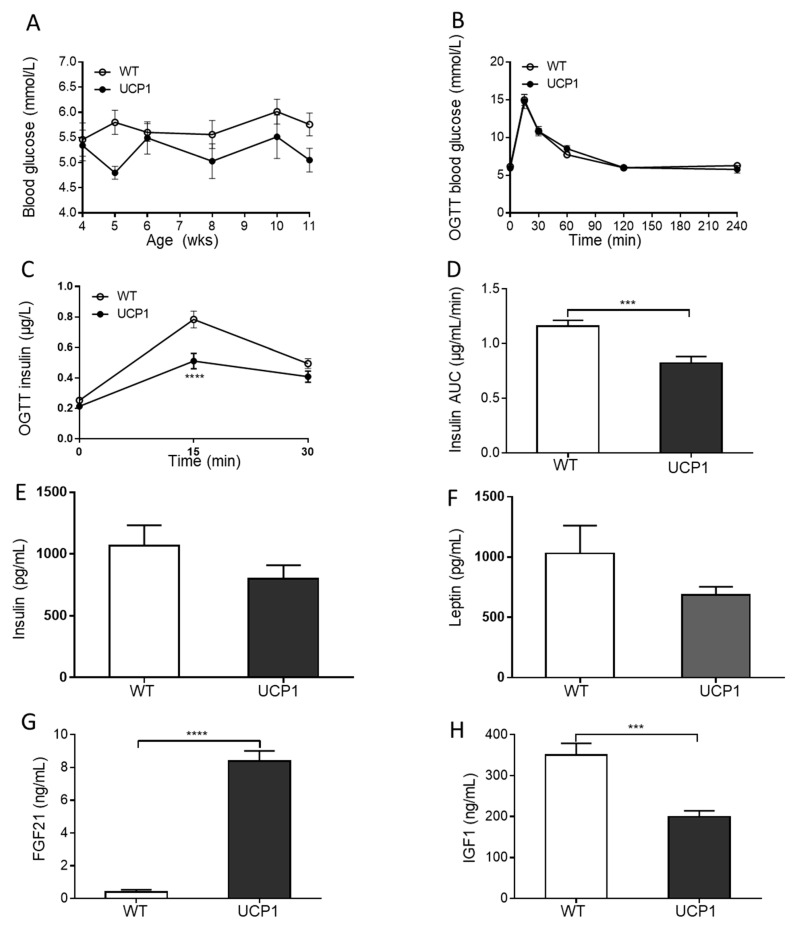
Metabolic health related parameters of UCP1-TG (UCP1) and WT mice. Blood glucose levels from the age of 4 until 11 weeks (**A**), glucose response to an oral glucose tolerance test (OGTT) at the age of 10 weeks (**B**), insulin response to the OGTT at the age of 10 weeks (**C**), incremental area under the curve (AUC) of the insulin response to the OGTT (**D**), plasma insulin concentrations (pg/mL) (**E**), plasma leptin concentrations (pg/mL) (**F**), plasma fibroblast growth factor 21 (FGF21) concentrations (ng/mL) (**G**) and plasma insulin-like growth factor 1 (IGF1) concentrations (ng/mL) (**H**). Data are expressed as mean +/− SEM (*n* = 10); (**A**–**C**): two-way ANOVA; (**D**–**H**): Student’s t-test, *** *p* < 0.001, **** *p* < 0.0001.

The health status of the animals was assessed by the analysis of random daytime blood glucose levels between week 4 and week 11 pp and by performing an oral glucose tolerance test (OGTT) in week 10 pp. UCP1-TG mice showed random blood glucose concentrations within the normal range, comparable to WT mice (Figure 3A). No difference was observed in the blood glucose response during the OGTT between UCP1-TG and WT mice (Figure 3B). However, the insulin response during the OGTT was significantly lower in UCP1-TG mice compared to WT littermates, indicative of improved insulin sensitivity in the UCP1-TG mice (Figure 3C,D) as reported previously for male mice [33,35]. In full agreement with the OGTT data, fasting insulin concentrations did not differ between the two groups at sacrifice (Figure 3E). Leptin levels also did not differ significantly between the WT and UCP1-TG group (Figure 3F), while the UCP-TG mice had higher plasma FGF21 levels (Figure 3G) and lower IGF1 concentrations (Figure 3H) at the time of sacrifice (Figure 3F), as previously described for male UCP1-TG mice [37].

### 2.4. Follicular Development in UCP1-TG Mice

**Figure 4:**
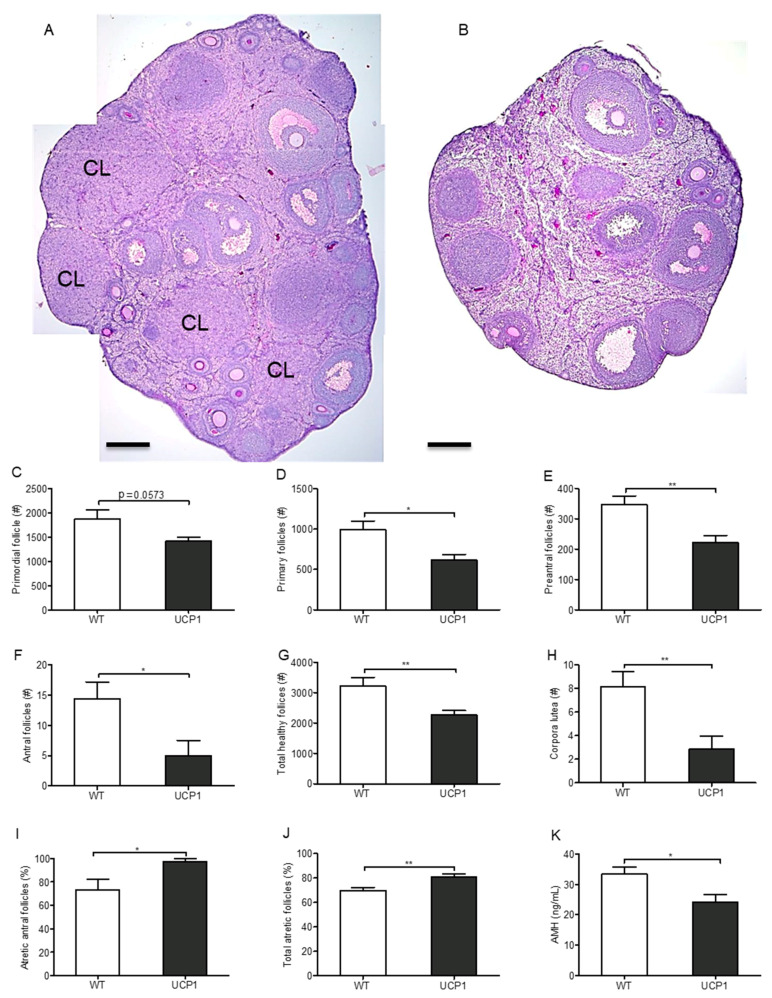
Ovarian follicular development of UCP1-TG (UCP1) and WT mice. Representative images of the gross ovarian morphology of WT (**A**) and UCP1-TG (**B**) mice. Numbers of primordial follicles (**C**), primary follicles (**D**), preantral follicles (**E**), antral follicles (**F**), total number of healthy follicles (**G**), and corpora lutea (**H**) per ovary. Percentage of atretic cleaved caspase 3 (cCASP3) positive antral follicles of WT and UCP1-TG mice (**I**) and percentage of total atretic follicles (of (pre)antral and unknown origin) (**J**). Plasma anti-Müllerian hormone (AMH) levels (ng/mL) (K). #, numbers; %, percentage; Values represent means +/− SEM 686 (*n* = 8, except for cCASP3, *n* = 4); (**C**–**K**): Student’s t-test * *p* < 0.05; **, *p* < 0.01. CL, corpus luteum, scale bar represents 200 μm.

Next, we investigated the effect of muscle-driven pseudo-starvation and the consequently occurring energy drain towards SKM tissue, on ovarian follicle development. Total ovarian follicle numbers were determined in UCP1-TG mice and compared to WT littermates (Figure 4A,B). The numbers of primary (Figure 4D, Figure S1A), preantral (Figure 4E, Figure S1B) and antral follicles (Figure 4F, Figure S1D) were all significantly reduced in the UCP1-TG mice compared to WT mice, while a downward trend was observed for the primordial follicle number (Figure 4C). Not surprisingly, the total number of healthy follicles (Figure 4G) was significantly lower in the UCP1-TG mice, while the percentage of atretic cCASP3 positive antral follicles (Figure 4I, Figure S1G) as well as the total percentage of atretic follicles (preantral, antral and of unknown origin, Figure 4J) was significantly higher compared to the WT littermates. Furthermore, if only early stage atretic follicles were analyzed in the UCP1-TG mice, a clear increasing trend in the percentage of atretic preantral follicles and the total percentage of atretic preantral and antral follicles was observed compared to the WT mice (Figure S2). In line with these observations, the number of corpora lutea per ovary was decreased (Figure 4H) in the UCP1-TG mice compared to the WT littermates. A complete absence of corpora lutea (CLs) was observed in around 40% (3/8) of the ovaries of the UCP1-TG females, as shown in Figure 4B, implicating that ovulation rate was severally affected by the energy drain towards SKM tissue. Whether this also leads to a reduced pregnancy rate and litter size was not investigated in the present study.

Plasma anti-Müllerian hormone (AMH) concentrations, a marker for the growing follicle pool (primary, preantral and antral follicles) [38], were significantly decreased in the UCP1-TG group (Figure 4K), fully in agreement with the histological analysis.

### 2.5. Follicular Development in FGF21^–/–^Mice

**Figure 5:**
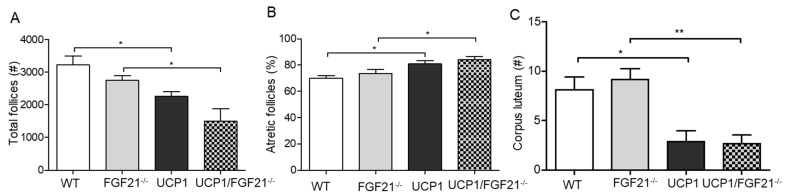
FGF21 and ovarian follicular development. Numbers of healthy follicles (**A**), percentage of atretic follicles (**B**), and numbers of corpora lutea (**C**) in WT (white), FGF21 knock out (FGF21^–/–^; grey), UCP1-TG (UCP1; black) and FGF21^–/–/^UCP1-TG (FGF21^–/–^/UCP1) mice (mixed). Data are expressed as mean +/− SEM (*n* = 6–8); #, numbers; %, percentage; (**A**–**C**): one-way ANOVA; *, *p* < 0.05; **, *p* < 0.01.

To determine whether the impaired ovarian follicular development in the UCP1-TG mice was caused by increased FGF21 levels, we investigated the follicle content in FGF21^–/–^ and UCP1-TG/FGF21^–/–^ mouse models. No circulating FGF21 could be detected in FGF21^–/–^ and UCP1-TG/FGF21^–/–^ mice, while plasma FGF21 levels underwent an approximately 16-fold increase in the UCP1-TG compared to the WT mice (Figure S3A). No differences in total follicle number, the percentage of atretic follicles or CL number per ovary were detected between UCP1-TG/FGF21^–/–^ mice and UCP1-TG mice (Figure 5A–C). In addition, no differences were seen in primordial, primary, preantral and antral follicle numbers between UCP1-TG/FGF21^–/–^ mice and UCP1-TG mice (Figure S4). At the same time, the total follicle numbers in the UCP1-TG/FGF21^–/–^group were significantly lower compared to the FGF21^–/–^ group (Figure 5A). Concomitantly, a significantly higher percentage of atretic follicles was observed in the UCP1-TG/FGF21^–/–^group compared to the FGF21^–/–^ group (Figure 5B). The total number of CLs per ovary in the UCP1-TG/FGF21^–/–^group was significantly lower compared to the FGF21^–/–^ group (Figure 5C). Finally, no significant differences in total healthy follicle numbers, atretic follicle numbers or CLs could be observed between WT and FGF21^–/–^ mice (Figure 5A–C). Together, these results indicated that the impaired follicular development observed in UCP1-TG mice was not due to the increase in circulating FGF21 levels in this transgene.

### 2.6. IGF1 Concentrations and Ovarian Follicle Numbers

**Figure 6:**
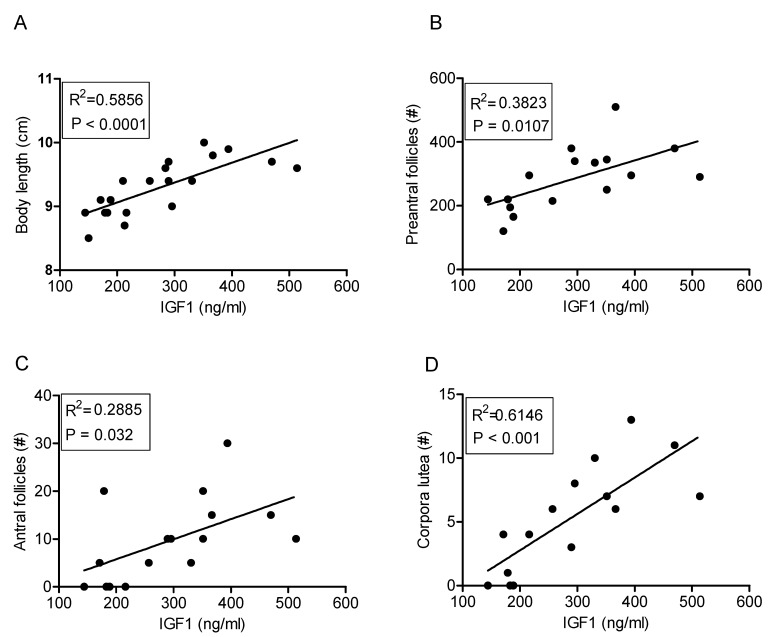
Correlation of plasma IGF1 concentrations with body length and parameters of ovarian follicular development in WT and UCP1-TG (UCP1) mice. Correlations of IGF1 with body length (**A**), number of preantral follicles (**B**), number of antral follicles (**C**) and number of corpora lutea (**D**); *n* = 8–10.

To determine whether the decrease in circulating IGF1 concentrations correlated with the morphometric measurements and ovarian phenotypic changes, linear regression analyses were performed. These analyses showed that plasma IGF1 concentrations were significantly correlated with body length (Figure 6A), preantral and antral follicle numbers (Figure 6B,C), and CL numbers (Figure 6D), indicating that the reduced IGF1 levels in the UCP1-TG mice may play a role in the impaired ovarian follicular development in the UCP1-TG mice.

### 2.7. Effects of UCP1-TG on Phosphorylated IRS1, AKT and IRS2 Expression in Granulosa Cells of Antral Follicles

**Figure 7:**
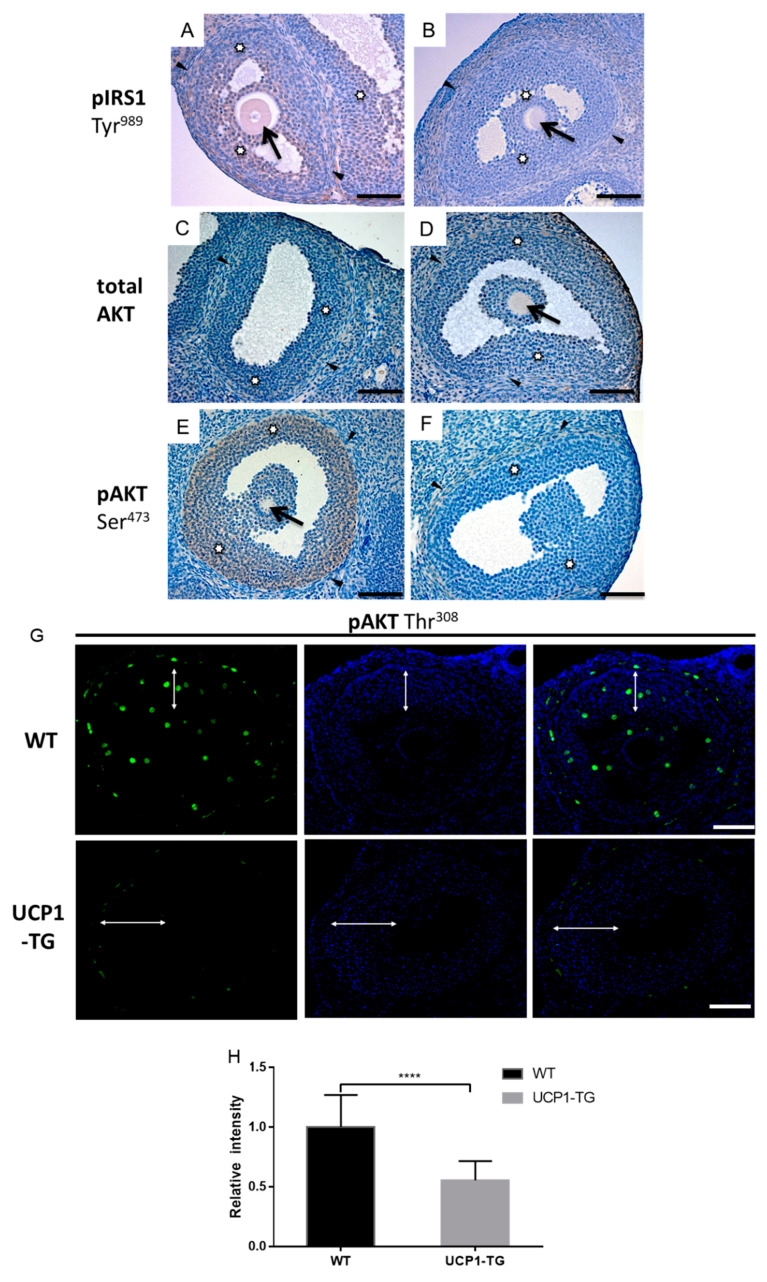
Immunostaining for phosphorylated IRS1, total and phosphorylated AKT Ser473 (brown staining) as markers of IGF1 signaling in healthy antral follicles of WT (**A**,**C**,**E**) and UCP1-TG (**B**,**D**,**F**) mice; immunofluorescence staining for phosphorylated AKT Thr^308^ in WT and UCP1-TG (**G**,**H**). Representative immunostaining of IRS1 phosphorylated at Tyr989 (**A**,**B**), total AKT (**C**,**D**), AKT phosphorylated at Ser473 (**E**,**F**) and AKT phosphorylated at Thr308, green, Thr308; blue, DAPI(**G**). Phosphorylation of IRS1 and AKT Ser473 is present in the granulosa cells of healthy antral follicles in WT ovaries but faint to absent in UCP1-TG mice. Similarly, immunofluorescence analysis showed the significantly decreased intensity of phosphorylated AKT Thr^308^ in the granulosa cells of healthy antral follicles in UCP1-TG mice in comparison to WT (**H**). (**H**): Student’s t-test, ****, *p* < 0.0001; total AKT immunostaining is moderate in the granulosa cells of WT animals and UCP1-TG mice. There was no difference in theca and stromal cell staining between the WT and UCP1-TG. Inserts show a detail of the granulosa layer. Arrow—oocyte, asterisk (**A**–**F**) and white two-sided arrow (**G**)—granulosa cells, arrowheads—theca cells. Scar bar represent 50 μm.

To substantiate the role of IGF1 in the ovarian follicular development of UCP1-TG mice, we performed immunohistochemical staining on the activation state of AKT, IRS1 and IRS2, key intermediates in IGF1 signaling. The staining was analyzed in healthy antral follicles and compared between WT and UCP1-TG mice. In the WT, ovary granulosa cells of healthy antral follicles showed moderate to strong pIRS1(Tyr989) and pAKT (Ser473) immunostaining (Figure 7A,E), while tAKT staining was somewhat less strong (Figure 7C). In the UCP1-TG, ovary granulosa cell pIRS1 and pAKT (Ser473) immunostaining was faint to absent (Figure 7B,F), while tAKT immunostaining in the UCP1-TG ovary was comparable to the WT ovary (Figure 7C,D). Similarly, immunofluorescence staining showed that pAKT (Thr^308^) was mainly observed in granulosa cells (Figure 7G). The quantification of the immunofluorescence showed that pAKT (Thr^308^) immunofluorescence intensity was decreased in the granulosa cells of healthy antral follicles in UCP1-TG mice in comparison to WT mice (Figure 7H). No differences in theca cell immunostaining were observed between the two groups (Figure 7). The same pattern of immunostaining was observed in atretic antral follicles (data not shown). IRS2 and pIRS2(Ser731) immunostaining was faint to absent in granulosa and theca cells of healthy antral follicles in both WT and UCP1-TG ovaries (Figure S5A–D). As was the case for pIRS1 staining, pIRS2 immunostaining in atretic antral follicles was not different from healthy follicles (data not shown).

## 3. Discussion

Female UCP1-TG mice show increased energy expenditure, a reduced body size, maintained adiposity and leptin levels, and reduced IGF1 levels, all similar to their male counterparts [32,39]. Importantly, the energy drains towards SKM tissue in these mice impairs ovarian follicular development, as UCP1-TG mice have significantly reduced primary, preantral and antral follicle numbers as well as CL numbers, while the percentage of atretic follicles is notably increased. Circulating IGF1 levels are significantly correlated with antral follicle numbers as well as CL numbers. The reduction in pIRS1 and pAKT immunostaining intensity in the granulosa cells of antral follicles in UCP1-TG mice suggests that IGF1 signaling is mediated by IRS1 and that the decrease in IGF1 levels seems to be associated with the female ovarian phenotype of the UCP1-TG mice.

The effects of the ectopic overexpression of the UCP1 gene on morphometric and metabolic parameters show a partial age-dependent pattern. Similar to the 12-week-old female mice of this study, body weight, absolute and relative lean body mass of 9- to 14-month-old female UCP1-TG mice is reduced compared to the WT littermate controls [12]. At the age of 12 weeks, no difference between UCP1-TG and WT controls in body temperature and absolute body fat content is seen, while relative fat mass is increased. In contrast, in 9- to 14-month-old UCP1-TG female mice body temperature and absolute and relative body fat content are decreased compared to WT mice [12]. This may imply that with ageing, less energy is stored in adipose tissue but instead used to keep body temperature in abeyance as much as possible. Energy intake and energy expenditure are increased while activity is reduced in both TG age groups [12]. Taken together, these data suggest that the metabolic consequences of an energy drain towards the UCP1 overexpressing SKM tissue gradually increase with age.

Energy availability is closely associated with female reproductive functioning. Indeed, CR and excess exercise result in reduced female fertility [5,6,40,41,42]. However, reproductive competence is maintained as long as food intake is maintained, even under energetic demanding conditions such as high levels of exercise and cold exposure ([8] and references therein). Feeding studies show a rapid restoration of fertility, before the restoration of adiposity, upon ad libitum feeding after CR [9], suggesting that the primary driver of fertility is the availability of metabolic fuels, rather than adiposity. This makes sense from an evolutionary perspective [43], where a temporary halt in fertility makes energy available for maintenance, thus preserving viability and indirectly fertility. In the UCP1-TG mouse model of SKM pseudo-starvation, energy is drained towards the muscle [11,44] and not surprisingly, ovarian follicular reserve and ovulation rate is impaired, despite the maintenance of adiposity and leptin levels.

IGF1 is an important mediator of growth but also plays a role in the regulation of substrate metabolism. It is therefore not surprising that circulating IGF1 levels are correlated with body length as well as antral follicle number and CL number. These correlations suggest that decreased peripheral IGF1 levels, at least in part, may mediate the negative impact on ovarian follicular content in the UCP1-TG mice. This is in line with observations in growth hormone receptor/growth hormone binding protein knockout (GHR/GHBP-KO) mice, which are fertile, indicative of sufficient FSH and LH levels but have strongly reduced IGF1 levels as well as lowered preantral and antral follicle numbers, and an increased percentage of atretic follicles. The restoration of IGF1 levels in these mice led to a decrease in the percentage of atretic follicles and a concomitant increase in healthy antral follicle numbers per ovary [18].

The next question that arises is how a reduction in peripheral IGF1 levels can influence follicular development and ovulation rate. In vitro studies employing granulosa cells have shown that FSH-induced targeted gene expression is dependent on the presence of IGF1 [27]. IGF1R is expressed on granulosa cells independent of the stage of follicular development and, upon the binding of IGF1, is responsible for the activation of IGF1 signaling [17]. The activation of IGF1 signaling in the ovary is a complex process that has at present only been partially unraveled, with IRS1 and IRS2 both being implicated as mediators of IGF1 signaling. Burks et al. [30] identified IRS2, a component just downstream of the insulin receptor and the IGF1R signaling cascade, as an essential integrator of female reproduction and energy homeostasis. In line with this, whole body knockout of *Irs2* resulted in the disruption of the oestrus cycle in mice. Histological analysis of the ovaries of these mice revealed a significant reduction in the number of antral follicles per ovary and a nearly complete absence of CLs. Neganova et al. [45] addressed the role of IRS2 in ovarian physiology in more detail. These authors show that the deletion of the *Irs2* gene in the central nervous system and hypothalamus, with normal expression levels in the pituitary, has minimal effect on reproductive function in mice, implicating the work by Burks et al. [30], who reported that the ovarian phenotype is indeed most likely due to the effects of the KO on pituitary function. The role for IRS2 in IGF1/insulin signaling and thus ovarian follicle development is further questioned by the absence of a change in IRS2 and pIRS2(Ser731) immunostaining in UCP1-TG mice, despite a reduction in pAKT immunostaining, as observed in the present study.

More recently, in a series of very elegant in vitro studies, Law and colleagues have shown that the binding of IGF1 to IGF1R is not sufficient to activate the PI3K/AKT pathway in rat granulosa cells [31,46]. The activation of PI3K/AKT depends on the phosphorylation of IRS1Y*M*X*M motifs by activated IGF1R as well as the activation of PKA through the binding of FSH to its receptor. Granulosa cells in vitro in the absence of FSH secrete constitutively sub-threshold concentrations of IGF1, which partially activate the IGF1R, though this activation is not sufficient to induce the phosphorylation of IRS1Y*M*X*M motifs [31]. These authors further showed that the ability of IGF1R to phosphorylate these IRS1 motifs is inhibited by the phosphorylation of IRS1 on at least four Ser residues (Ser318, Ser346, Ser612, Ser789) [46]. The de-phosphorylation of these Ser residues needs to occur in order for IRS1Y*M*X*M phosphorylation to proceed. The binding of FSH to its receptor and the subsequent activation of PKA promotes the activation of the β-catalytic subunit of protein phosphatase 1 (PP1cβ) that forms a complex with its regulatory subunit myosin phosphatase target subunit 1 (MYPT1), phosphorylating MYPT1 on Ser668. This leads to the activation of PP1cβ and the subsequent de-phosphorylation of the four Ser residues on IRS1, facilitating the phosphorylation on Tyr989, a canonical Tyr*XXMet motif, consequently activating PI3K and causing the phosphorylation of AKT on Ser473 and Thr308, thus activating AKT [47]. Direct in vivo evidence for these in vitro observations is not yet available. The concentrations of IGF1 in follicular fluid and plasma are substantially higher than the amount of IGF1 secreted by granulosa cells in vitro. However, one has to keep in mind that the concentrations of IGF binding proteins greatly exceed IGF1 concentrations in the circulation and follicular fluid, suggesting that there is very little free IGF1 available to activate the IGF1R [47]. Law and colleagues therefore hypothesized that it is the granulosa cell-derived IGF1 that, together with FSH, activates the PI3K/AKT pathway [47]. Our immunohistochemical data also show that in WT mice in vivo, in the presence of FSH and systemic and locally produced IGF1, IRS1 is phosphorylated on Tyr^989^ and that concomitantly AKT is phosphorylated on Thr308 and Ser473in granulosa cells, as postulated by Law et al. [47]. However, in the UCP1-TG mice, where systemic IGF1 concentrations are reduced by approximately 40%, granulosa cell pIRS1(Tyr989) and pAKT(Thr308)/pAKT(Ser473) immunostaining becomes faint to absent, indicative of reduced activation. These data suggest that changes in plasma IGF1 levels may influence the activation of the IGF1R signal transduction pathway, as was also reported in the GHR/GHBP KO mice [18], underscoring the important role of systemic IGF1 in ovarian follicle development. We therefore hypothesize that the reduction in follicle numbers and ovulation rate in the UCP1-TG mouse is associated with the reduced availability of systemic IGF1 and the subsequent reduced activation of the IGF1R/PI3K/AKT pathway. These observations further seem to question the hypothesis by Law and colleagues, that systemic and follicular fluid IGF1, due to its binding to IGF binding proteins, is of no influence on the activation of the IGF1R-PI3K/AKT pathway in granulosa cells. Whether the functional status of IRS1 and AKT in granulosa cells of antral follicles in UCP-TG mice can be reversed by exogenous IGF1 supplementation needs to be further explored. Data from previous studies in GH/GHBP-KO mice with severely reduced systemic IGF1 levels suggest that at least a partial reversal of the ovarian phenotype occurs upon IGF1 treatment [18].

UCP1-TG mice also display alterations in several other plasma hormones, including strongly increased levels of fibroblast growth factor 21 (FGF21) [37]. While the liver is regarded to be the primary contributor to circulating FGF21 levels [48], the elevated plasma FGF21 concentrations in UCP1-TG mice have been shown to arise from SKM [37]. Studies addressing the effects of FGF21 on female reproduction are limited and the results somewhat contradictory. FGF21 overexpression causes infertility in female mice fed a chow diet [48,49], but when these mice are fed a high fat diet, fertility is restored without altering circulating concentrations of FGF21 [50]. Our data show that elevated levels of FGF21 are not likely the cause of the ovarian phenotype in the UCP1-TG, as the deletion of the *Fgf21* gene does not have an effect of ovarian follicle content, atresia or ovulation rate. This assumption is further supported by the fact that neither the ovarian phenotype nor circulating IGF1 levels differ between the UCP1-TG and the UCP1-TG/FGF21 KO mice.

Next to FGF21, the UCP1-TG mice also have severely increased levels of growth differentiation factor 15 (GDF15), due to the transgene-induced overexpression of the *Gdf15* gene in SKM [36]. Whether these more than 10-fold increased levels of GDF15 are responsible for the observed effects on the UCP1-TG ovary, including a trend in reduced primordial follicle number remains to be elucidated.

## 4. Materials and Methods

### 4.1. Chemicals and Antibodies

All chemicals were purchased from Sigma (Zwijndrecht, the Netherlands) unless indicated otherwise. Antibodies against phospho-AKT serine473 (pAKT (Ser473), lot no.19, cat. no. 4060), pAKT(Thr^308^) (lot no. 22, cat. no. 9275), and cleaved caspase 3 (cCASP3, lot no 37, cat. no. 9661) were purchased from Cell Signaling Technology (Leiden, The Netherlands). Antibodies against total AKT (tAKT, lot nr GR59504, cat no. ab8805), pIRS2(Ser731) (lot no.GR211245-1, cat. no. ab3690) and IRS2 (lot no. GR219213-2, cat. no. ab134101) were purchased from Abcam (Cambridge, UK). The antibody against pIRS1(Tyr989) (lot no. H1115, cat. no. 17200-R) was obtained from Santa Cruz (Santa Cruz Biotechnology, Heidelberg, Germany).

### 4.2. Animals

The animal experiments were approved by the ethics committee of the Ministry of Agriculture and Environment (State Brandenburg, Germany, permission number GZ V3-2347-16-2013, 05-09-2013) and carried out in accordance with the EU Directive 2010/63/EU for animal experiments.

Experiments were performed in female UCP1-TG mice and wild-type (WT) littermate controls on a C57Bl/6- background. UCP1-TG mice [12] and *Fgf21*-knockout (FGF21^–/–^) mice [51] were generated as described previously. UCP1-TG mice were crossed with FGF21^–/–^ mice to produce four genotypes of mice: wild-type (WT), FGF21^–/–^, UCP1-TG, and UCP1-TG/FGF21^–/–^ mice [44]. The UCP1 transgene is specifically overexpressed in skeletal muscle under the control of the 2.2-kb human skeletal actin promoter fragment, which confers striated muscle-specific gene expression, with little or no ectopic expression in other tissues [12,52,53]. Mice were group-housed at 23 °C and exposed to a 12:12 h dark–light cycle with ad libitum access to a chow diet (Ssniff A153F0300, Soest, Germany) and water. Ten WT and 10 UCP1-TG female mice were dissected in the morning 2 h after food withdrawal at 12 weeks of age, after which blood and tissue samples were collected.

### 4.3. Anthropometric and Metabolic Assessments

Body length was measured as the length between nose and anus. Body composition was measured by quantitative magnetic resonance (QMR, EchoMRI 2012 Body Composition Analyzer, Houston, TX, USA) [44]. Indirect calorimetry was performed in a Phenomaster system (TSE Systems GmbH, Bad Homburg, Germany) to calculate energy intake, energy expenditure, with the parallel measurement of spontaneous physical activity using infrared detection (TSE Systems GmbH, Germany), at the age of 11 weeks, as described previously [15]. Body temperature was measured at 11 weeks of age using a BAT-12 Microprobe thermometer (Physitemp, Clifton, NY, USA).

Random blood samples were taken from the tail vein to determine blood glucose levels at the age of 4, 5, 6, 8, 10 and 11 weeks using a Contour glucose sensor (Bayer, Leverkusen, Germany). At the age of 10 weeks an oral glucose tolerance test (OGTT) was performed; for this, 2 mg of glucose per g body weight was applied by oral gavage 2 h after food withdrawal. Blood glucose levels were measured before, and 15, 30, 60, 120, and 240 min after glucose application. Plasma insulin concentrations were measured before, and 15 and 30 min after glucose application by an ultra-sensitive ELISA assay according to the manufacturer’s instructions (EIA-3440, DRG Instruments GmbH, Marburg, Germany). The inter-and intra-assay variation for the insulin ELISA was 6%.

### 4.4. Plasma IGF1, AMH and Leptin Measurements

IGF1 values (plasma diluted 1:5; mouse/rat IGF 1 Quantikine ELISA Kit; R&D Systems, Cat# MG100, sensitivity 8.4 pg/mL), anti-Müllerian hormone (AMH) values (plasma diluted 1:4; AMH Gen II ELISA Kit, Beckman Coulter, A79765, Sinsheim, Germany, sensitivity 10 pg/mL) and leptin levels (plasma diluted 1:5; Bio-Plex Pro mouse diabetes assay, Biorad, Veenendaal, The Netherlands) were determined according to the protocol of the respective suppliers as reported previously. The inter-and intra-assay variation for the IGF1 ELISA was 7% and for the AMH assay and 9% for the leptin assay [44,51,54]. All the ELISA kits used in this study have been tested in mouse/rat previously as indicated by the manufacturers.

### 4.5. Histological Evaluation of Ovarian Tissues

The right ovaries of 6 to 8 animals were fixed in diluted Bouin’s fluid (0.9% picric acid, 4% formaldehyde, 5% glacial acetic acid) for 24 h at 4 °C and embedded in paraffin. The left ovaries of the animals were fixed in 4% formalin for 24 h at 4 °C and embedded in paraffin. The Bouin’s fixed ovaries were serially sectioned at a thickness of 5 μm. Every fifth section of these ovaries was mounted on glass slides, stained with periodic acid Schiff’s reagent (PAS) and Mayer’s haematoxylin (Klinipath, Duiven, The Netherlands), and examined by light microscopy at a 5x to 40x magnification (Zeiss Axioscoop II microscope). From these sections, the numbers of healthy primordial, primary, preantral and antral follicles were counted as described previously [18,55,56]. Briefly, in every fifth ovarian section primary, preantral and antral follicles were counted when the oocyte with nucleus and nucleolus were present in the follicular cross section. The size of the nucleolus is smaller than 25 μm; in this way we prevented counting the same follicle twice. Primordial follicles were also counted every fifth section when the oocyte with multiple nucleoli was visible in the follicular cross section. The nucleus of primordial follicles is smaller than 25 μm and therefore there was, in contrast to the larger follicles, no risk of counting the same follicle twice. In order to estimate the total number of follicles within one ovary, the number of primordial, primary, preantral and antral follicles counted in the mounted sections was multiplied by five to account for the fact that every fifth section was used in the follicle counting [18,56,57].

Early atretic preantral follicles were recognized by the presence of a degenerating oocyte, disorganized granulosa cell layer with the presence of no or a few cleaved caspase 3 (cCAP3) apoptotic nuclei, while the surrounding theca cells showed clear signs of hypertrophy, as described previously [58]. Early antral follicles were considered to be atretic when more than 5% of the granulosa cells in the follicular cross section showed signs of apoptosis (presence of apoptotic bodies, cell fragments containing condensed chromatin) and stained positively for the presence of cCASP3. The surrounding theca layer of these follicles showed signs of hypertrophy. As atresia proceeded in antral follicles, the granulosa cells were lost completely, the oocyte degenerated and the antral space collapsed, leaving remnants of the zona pellucida and hypertrophied theca cells as described previously [59,60]. These follicles were scored as atretic follicles of unknown origin. In order to prevent the counting of the same atretic follicle more than once, an estimate of the percentage of atretic follicles was made as described previously [61].

To determine the number of corpora lutea (CLs) per ovary, complete overview pictures were taken of every tenth ovarian section using a Zeiss Axioscoop II microscope equipped with an MRc5 camera and Axiovision 4.8.0.0 software (Zeiss GmbH, Jena, Germany). By following the CLs throughout the ovary, it was possible to determine the total number of CLs per ovary.

### 4.6. Immunohistochemistry

To determine the presence of tAKT, pAKT(Ser473), pAKT(Thr308), pIRS1(Tyr989), pIRS2(Ser731), tIRS2, and cCASP3, immunohistochemistry was performed as previously reported [56,62,63] with minor modifications. Five-µm thick paraffin sections were cut from formalin fixed ovaries and mounted on Superfrost Plus glass slides (Menzel, Braunschweig, Germany). For each antibody, 3 randomly selected ovarian sections of, respectively, WT (*n* = 6) and UCP1-TG (*n* = 6) animals were simultaneously incubated in a single run according to the immunohistochemical protocol for the specific antibody. This made it possible to qualitatively and quantitatively determine possible differences in staining intensity between WT and UCP1-TG ovaries. The immunohistochemical staining procedure was repeated at least five times for each separate antibody. Briefly, sections were deparaffinized and rehydrated, after which epitope antigen retrieval with sodium citrate buffer (pH 6, 10 min) was performed in a microwave oven at 96 0C. Slides were cooled down to room temperature and subsequently rinsed in 0.01 M phosphate buffered saline pH 7.4 (PBS). Sections were pre-incubated with 10% (wt/v) normal goat serum in PBS for 30 min and incubated overnight at 4 °C with the respective primary antibodies (tAKT, diluted 1:500, pAKT(Ser473), diluted 1:200; pIRS1(Tyr989) diluted 1:100; pIRS2(Ser731), diluted 1:100; tIRS2, diluted 1:200; and cCASP3, diluted 1:1000 in PBS to which 0.05% acetylated bovine serum albumin (BSAc) (Aurion, Wageningen, The Netherlands) was added). The goat-anti-rabbit biotin labelled secondary antibody (Vector Laboratories, Burlingame, CA, USA) was diluted 1:200 (*v*/*v*), in PBS-BSAc. The avidin–biotin complex (ABC) was diluted 1:1000 (*v*/*v*) (Vectors stain kit elite, Vector Laboratories) in PBS-BSAc. Bound antibody was visualized using the 3-3′ diaminobenzidine kit (Immpact DAB, Vector Laboratories) at a dilution of 1:200 (*v*/*v*). Sections were counterstained with Mayer’s haematoxylin. For immunofluorescence staining, after primary antibody incubation (pAKT(Thr308), diluted 1:100) overnight at 4 °C, sections were rinsed with PBS and incubated in the dark with a secondary Alexa fluor 488 labeled goat-anti-rabbit antibody (A-11008, ThermoFisher Scientific, Waltham, MA, USA) diluted 1:200 (*v*/*v*) in PBS-BSA-c for 1 h at room temperature. Sections were counterstained with 4′,6-diamidino-2-phenylindole (DAPI) (0.5 μg/mL; Sigma) for 10 min. Sections were imaged at 20× magnification using a fluorescence microscope (Leica DM6B), a digital camera (DFC365 FX), and imaging software (LasX; all Leica Microsystems, Amsterdam, The Netherlands). The mean staining intensity of the granulosa cell layer was determined using ImageJ (http://rsbweb.nih.gov/ij/download.html accessed on 6 November 2020). Control sections were incubated with isotype IgG (Vector Laboratories) instead of the primary antibody according to the manufacturer’s instructions. Background staining was negligible in the control incubations.

### 4.7. Statistical Analysis

GraphPad Prism version 8.0 (Graphpad Software, San Diego, CA, USA) was used for statistical analysis, with Student’s t test being used to compare the two groups if data were normally distributed. Two-way ANOVA (repeated measures, matched values) followed by the Bonferroni post hoc test was used for body weight, body composition and OGTT in time analysis. The relations between plasma IGF1 concentrations and body length as well as ovarian follicle and corpora lutea numbers were analyzed using linear regression. Data were expressed as mean *+/*− SEM; *p* values < 0.05 were considered significantly different.

## 5. Conclusions

Taken together, our data show that the ectopic expression of UCP1 in SKM negatively affects ovarian follicle development and ovulation rate, a fact that is associated with a reduction in circulating IGF1 levels and a concomitant reduced immunostaining of IRS1-IP3K/AKT. Our data underscore the importance of IGF1 signaling not only in metabolic processes but also in normal ovarian follicular development and ovulation.

## Data Availability

All data generated or analyzed during this study are included in this article or in the data repositories listed in References.

## Supplementary Materials

The following are available online at https://www.mdpi.com/article/10.3390/ijms22073557/s1.
