# Supplementary material for: Pseudo-Starvation Driven Energy Expenditure Negatively Affects Ovarian Follicle Development
Source: Int J Mol Sci. 2021 Mar 30;22(7):3557. doi: 10.3390/ijms22073557 (PMC8036485; doi:10.3390/ijms22073557)
Supplement: Supplementary file 1 [file ijms-22-03557-s001.zip › Supplemental Figure/Meng et al Supplemental Figure 1.pptx]

## Slide 1
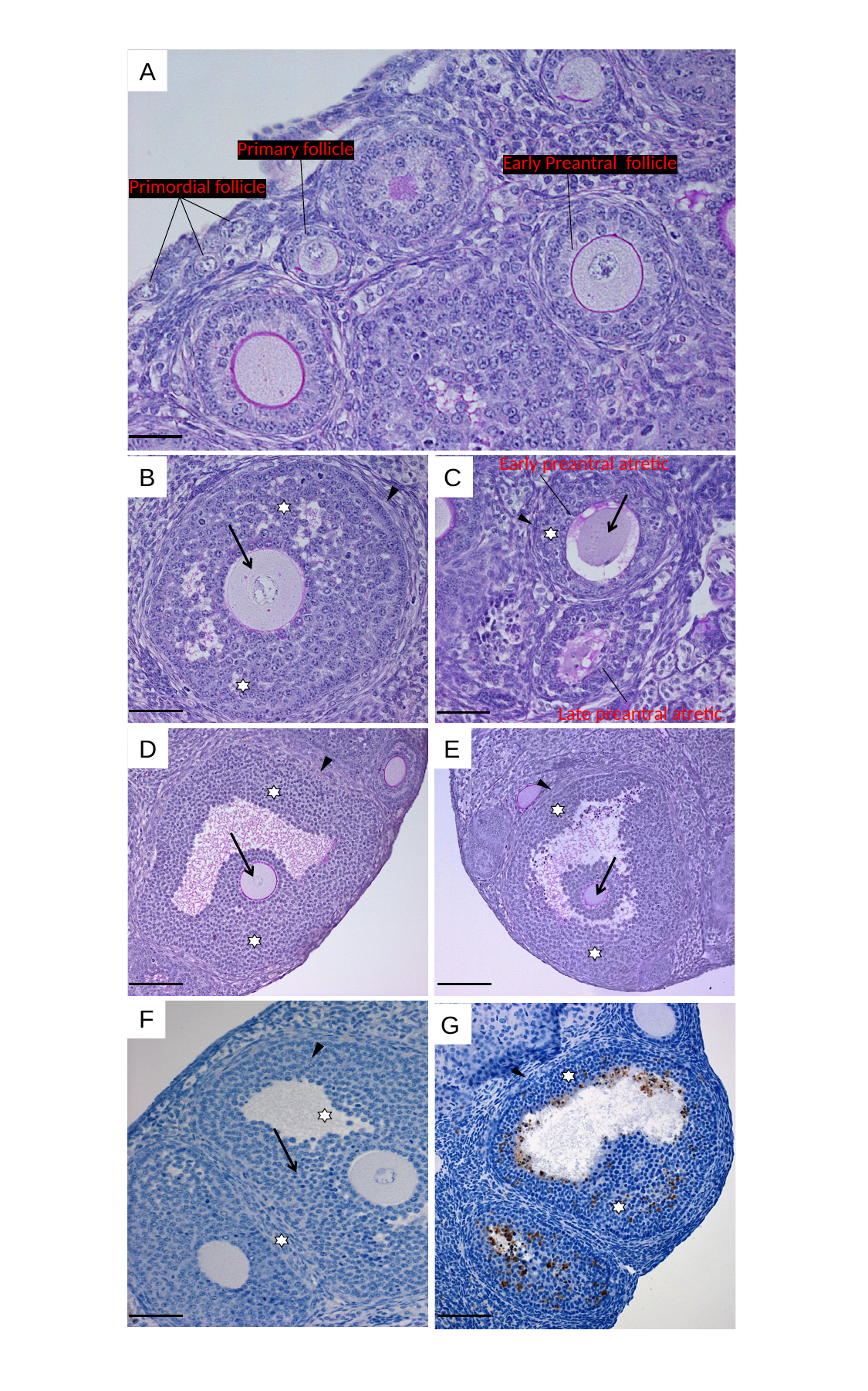

A
Primary follicle
Early Preantral follicle
Primordial follicle
Early preantral atretic
B
C
Late preantral atretic
D
E
F
G
