# Supplementary material for: Pseudo-Starvation Driven Energy Expenditure Negatively Affects Ovarian Follicle Development
Source: Int J Mol Sci. 2021 Mar 30;22(7):3557. doi: 10.3390/ijms22073557 (PMC8036485; doi:10.3390/ijms22073557)
Supplement: Supplementary file 1 [file ijms-22-03557-s001.zip › Supplemental Figure/Meng et al Supplemental Figure 4.pptx]

## Slide 1
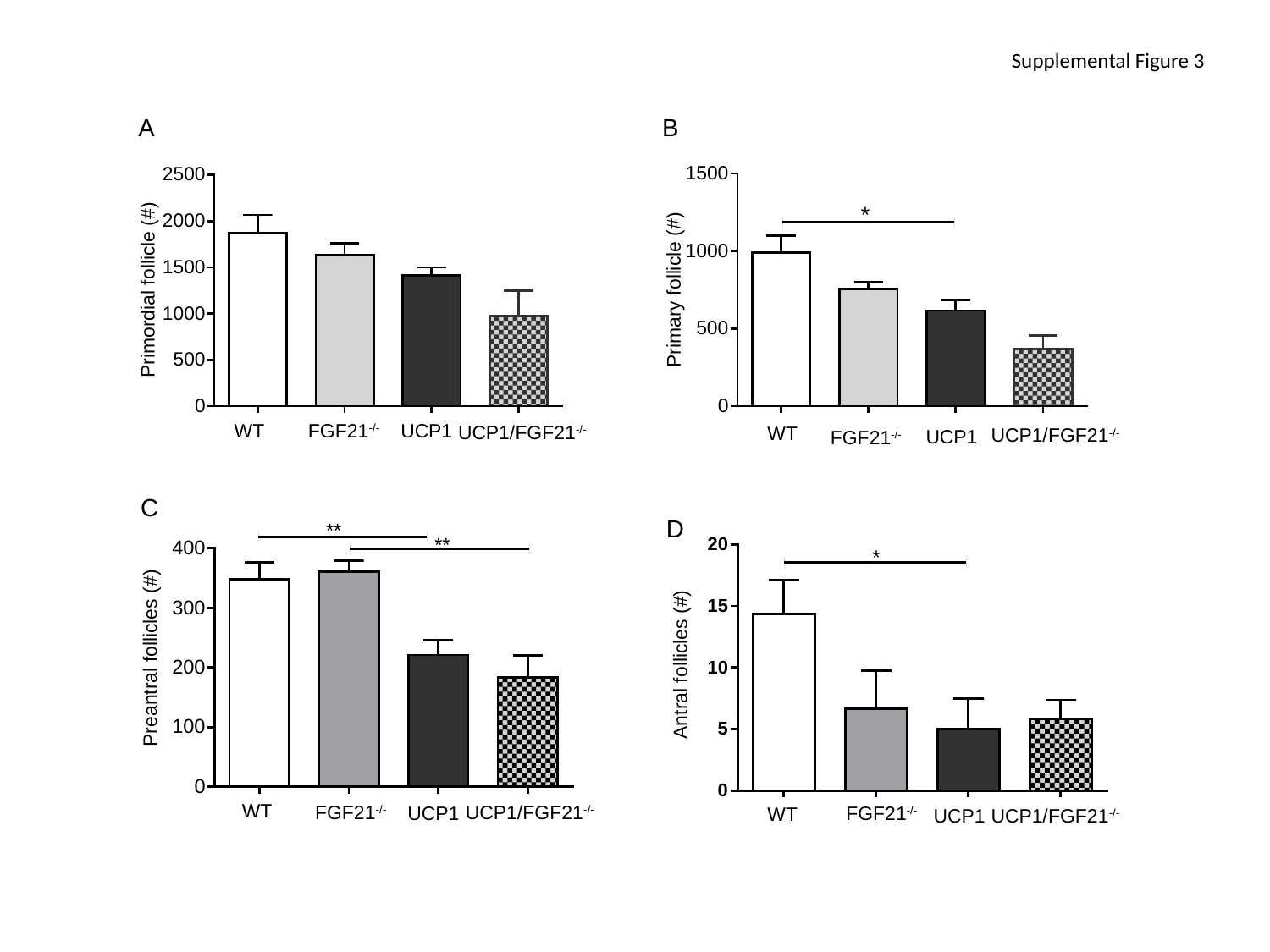

Supplemental Figure 3
A
B
Primordial follicle (#)
Primary follicle (#)
FGF21-/-
WT
UCP1
UCP1/FGF21-/-
WT
UCP1/FGF21-/-
UCP1
FGF21-/-
C
D
Preantral follicles (#)
Antral follicles (#)
WT
FGF21-/-
UCP1/FGF21-/-
FGF21-/-
UCP1
WT
UCP1
UCP1/FGF21-/-
