# Supplementary figures and images for: Pseudo-Starvation Driven Energy Expenditure Negatively Affects Ovarian Follicle Development
Source: Int J Mol Sci. 2021 Mar 30;22(7):3557. doi: 10.3390/ijms22073557 (PMC8036485; doi:10.3390/ijms22073557)

## Slide 1
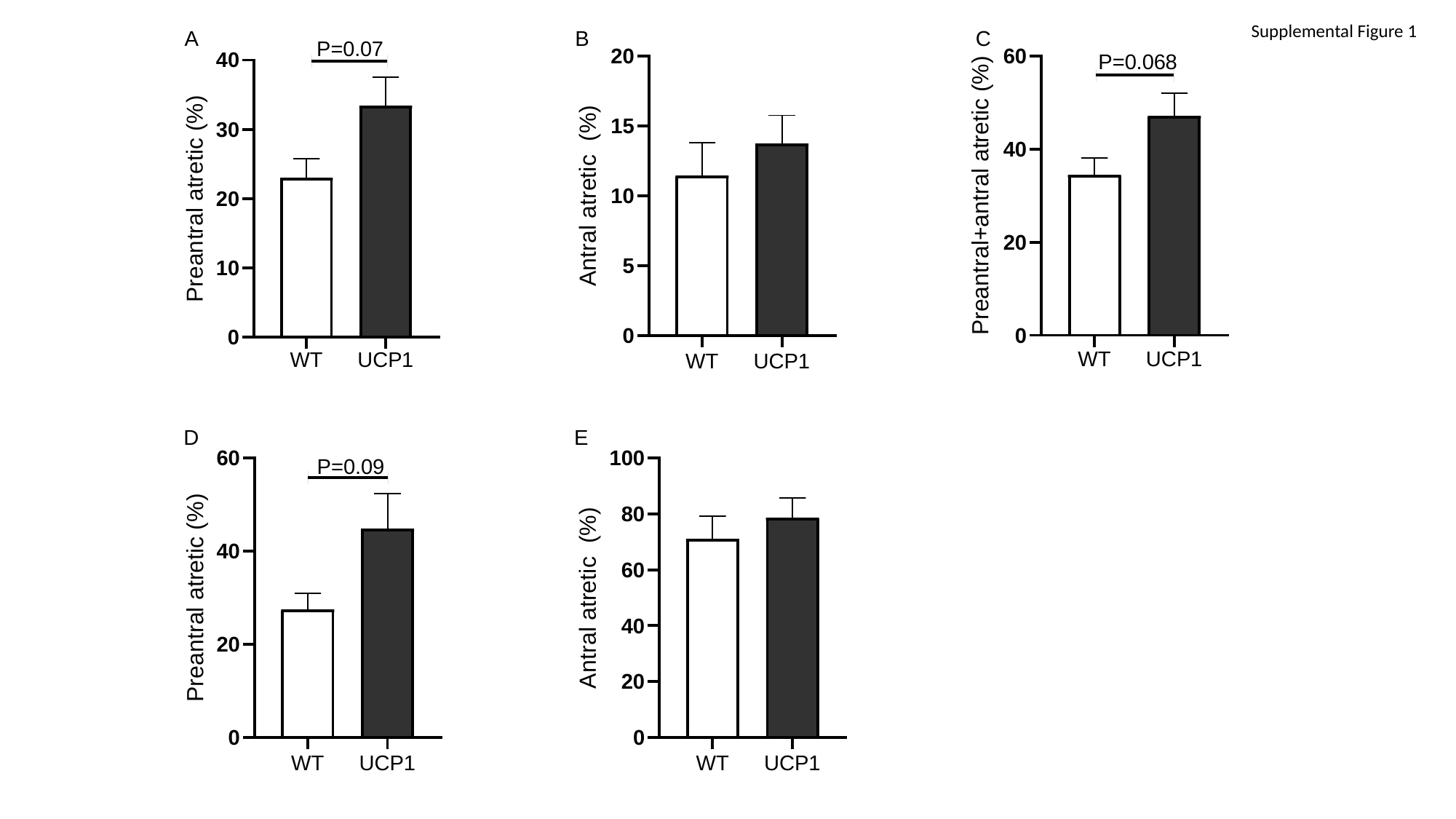

Supplemental Figure 1
B
C
A
D
E

Supplement: Supplementary file 1 [file ijms-22-03557-s001.zip › Supplemental Figure/Meng et al Supplemental Figure 2.pptx]

## Slide 1
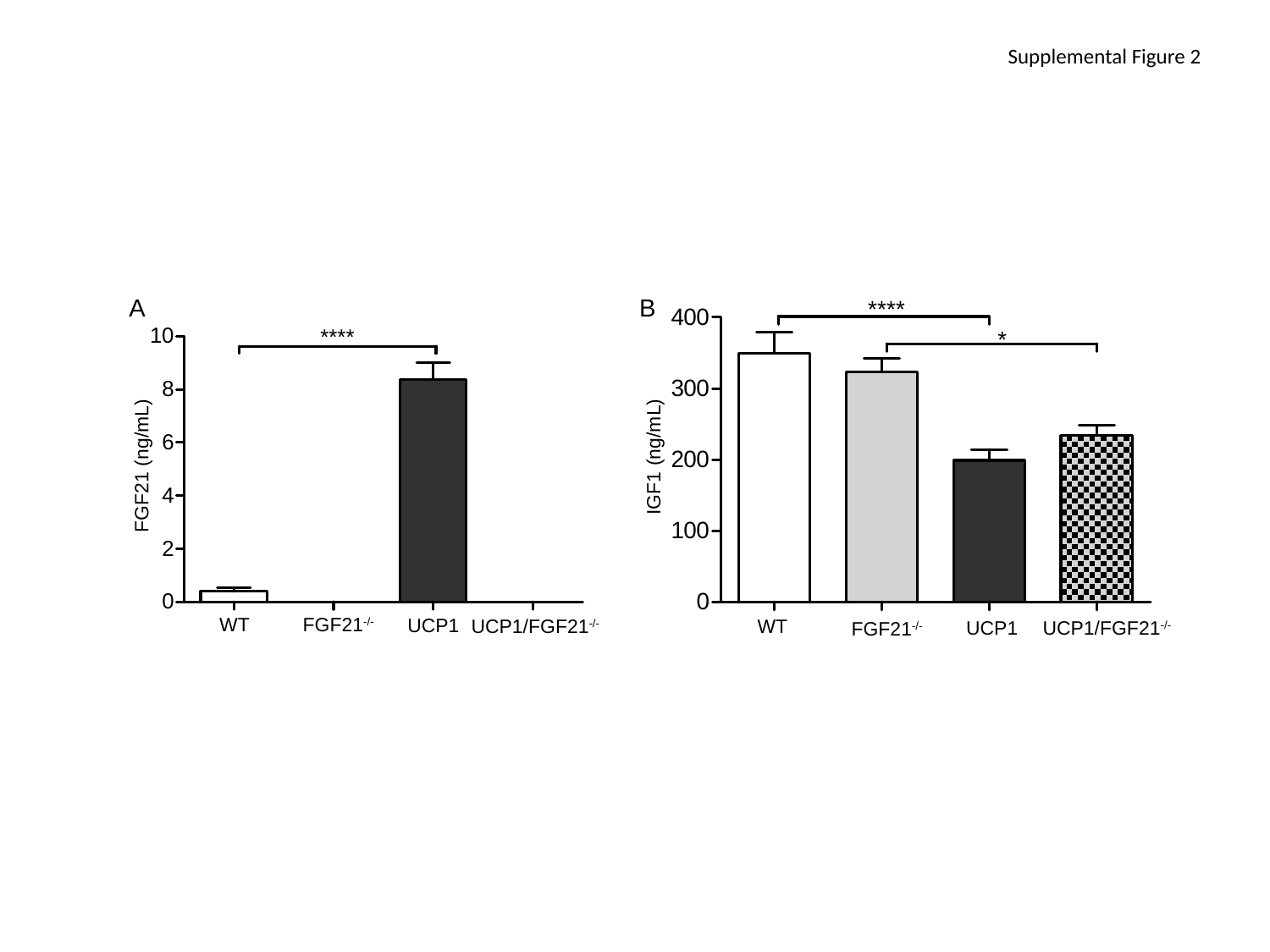

Supplemental Figure 2
B
A
IGF1 (ng/mL)
FGF21 (ng/mL)
WT
FGF21-/-
UCP1
UCP1/FGF21-/-
WT
UCP1/FGF21-/-
UCP1
FGF21-/-

Supplement: Supplementary file 1 [file ijms-22-03557-s001.zip › Supplemental Figure/Meng et al Supplemental Figure 3.pptx]

## Slide 1
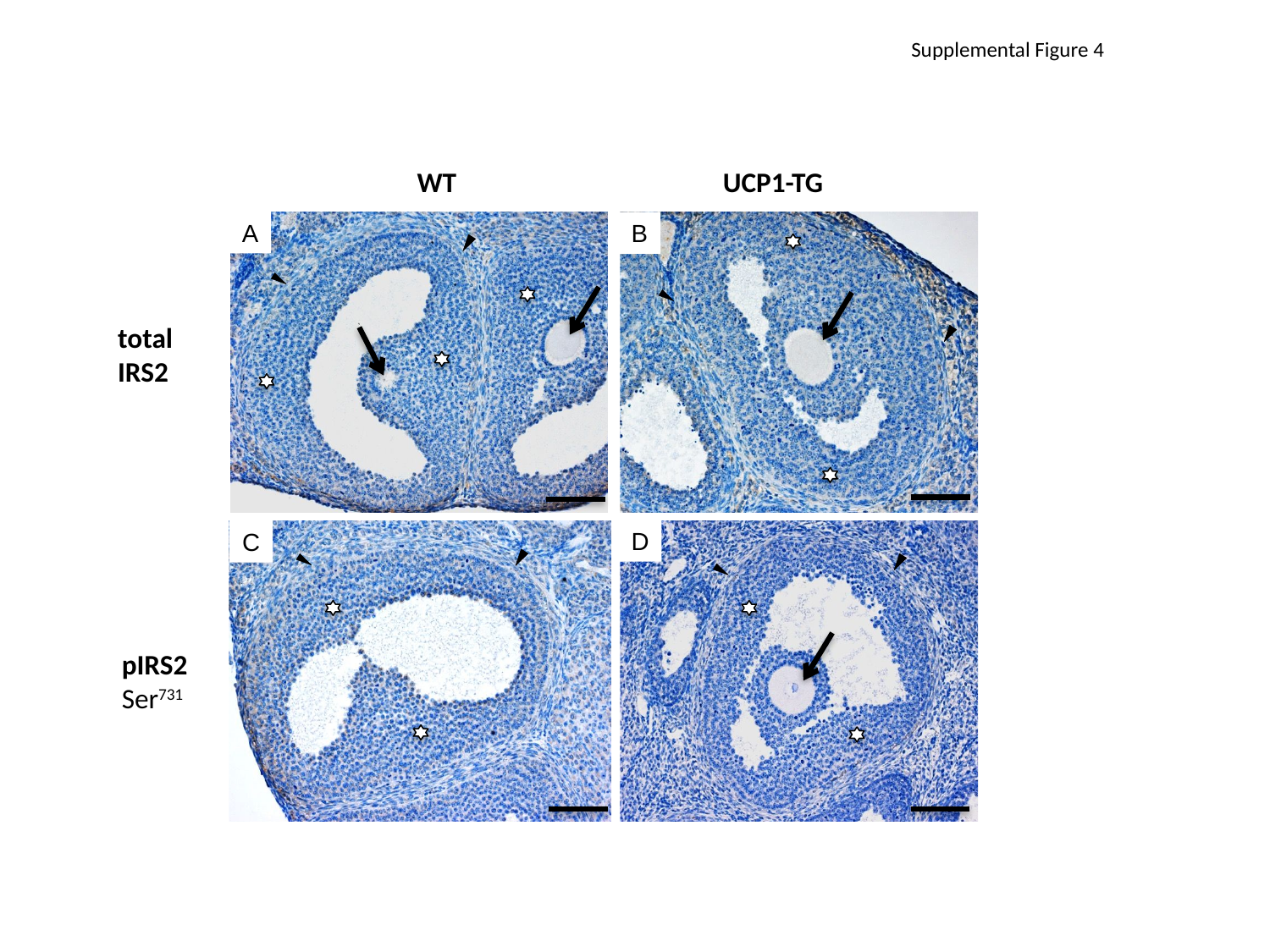

Supplemental Figure 4
# WT UCP1-TG
A
B
D
C
total IRS2
pIRS2
Ser731

Supplement: Supplementary file 1 [file ijms-22-03557-s001.zip › Supplemental Figure/Meng et al Supplemental Figure 5.pptx]
